# Tensional acoustomechanical soft metamaterials

**DOI:** 10.1038/srep27432

**Published:** 2016-06-06

**Authors:** Fengxian Xin, Tianjian Lu

**Affiliations:** 1 State Key Laboratory for Strength and Vibration of Mechanical Structures, Xi’an Jiaotong University, Xi’an 710049, P.R. China; 2 MOE Key Laboratory for Multifunctional Materials and Structures, Xi’an Jiaotong University, Xi’an 710049, P.R. China

## Abstract

We create acoustomechanical soft metamaterials whose response to uniaxial tensile stressing can be easily tailored by programming acoustic wave inputs, resulting in force versus stretch curves that exhibit distinct monotonic, s-shape, plateau and non-monotonic snapping behaviors. We theoretically demonstrate this unique metamaterial by considering a thin soft material sheet impinged by two counter-propagating ultrasonic wave inputs across its thickness and stretched by an in-plane uniaxial tensile force. We establish a theoretical acoustomechanical model to describe the programmable mechanics of such soft metamaterial, and introduce the first- and second-order tangential stiffness of its force versus stretch curve to boundary different behaviors that appear during deformation. The proposed phase diagrams for the underlying nonlinear mechanics show promising prospects for designing tunable and switchable photonic/phononic crystals and microfluidic devices that harness snap-through instability.

Metamaterials are man-made architecture materials that possess unusual and fantastic properties far beyond those of their compositions^1^,^2^. Significant advances in optical, acoustical and thermal metamaterials have inspired the study of mechanical metamaterials. Mechanical metamaterials with extraordinary properties can be developed via unusual elasticity tensor or mass-density tensor, such as negative compressibility^3^,^4^, negative Poisson’s ratio^5^,^6^,^7^,^8^,^9^,^10^,^11^,^12^, negative mass density and elastic modulus^13^, vanishing shear modulus^14^,^15^, and exceptional elastodynamical behaviors^16^,^17^,^18^,^19^,^20^. More recently, the construction of mechanical metamaterials by harnessing elastic instabilities has attracted much attention, for applications such as trapping elastic energy^21^, amplifying mechanical response^22^, programmable behavior^23^,^24^ and transforming wave propagation^25^. Nonetheless, most existing metamaterials have rather complicated artificial micro/nanoscale architectures or embedded inclusions, which raises naturally the question whether there exists one kind of metamaterial that can be constructed from a homogenous and isotropic medium such as soft material.

This work presents tensional acoustomechanical metamaterials by harnessing the snap-through instability of soft materials and demonstrates that, when subjected to varying ultrasonic wave inputs, these novel metamaterials exhibit phase switches and programmable mechanical responses. The current study is a first attempt to employ acoustic radiation forces generated by inputting acoustic waves to program the nonlinear mechanical behavior of soft materials, providing a new yet simple methodology for designing tunable and switchable optical, acoustical, thermal and mechanical metamaterials.

## Theoretical model

We carry out theoretical analysis on the programmable mechanical behaviors of tensional acoustomechanical soft metamaterials. An ultrasonic wave is capable of generating acoustic radiation stresses when it is propagating in a soft material, due to momentum flux transfer between material particles. By time-averaging the acoustic momentum flux **T**, the acoustic radiation stress tensor can be written as^26^,^27^,^28^,^29^,^30^,^31^:


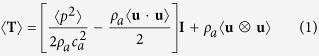


where *p* is the acoustic pressure, **u** is the particle velocity vector, **I** is the identity matrix, *ρ*_*a*_ and *c*_*a*_ is the material density and acoustic speed, respectively. Here, the soft material is taken as homogenous and isotropic with its bulk modulus much larger than its shear modulus so that, particularly at ultrasonic frequencies, the soft material bears negligible dynamical shear stress. Therefore, the soft material behaves like a fluid and its bulk modulus plays a dominant role in ultrasonic wave propagation. Note further that, because the frequency of ultrasonic wave is very high (>10^6 ^Hz), a material cannot response to such fast time-varying momentum transfer due to the hysteretic nature of typical materials. Rather, the material presents a static deformation response to effective momentum transfer (*i.e*., the steady acoustic radiation force expressed in the form of time-averaged momentum transfer flux).

As formulated in Eq. (1), the acoustical radiation stress scales as 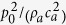, *p*_0_ being the amplitude of input sound pressure. As typical acoustic pressure lies between 0.1 and 4 MPa, the corresponding acoustic radiation stress varies between 70.25 kPa and 112 MPa in air, and between 4.44 Pa and 7.11 kPa in water. Such acoustic radiation stress is sufficient to induce large deformation in soft materials, for soft materials generally possess small shear moduli that range from dozens of times Pa to several times kPa. Thereby, acoustic radiation force can be comparable to mechanical force while much superior over the latter due to its non-contact and fast manipulation.

**Figure 1:**
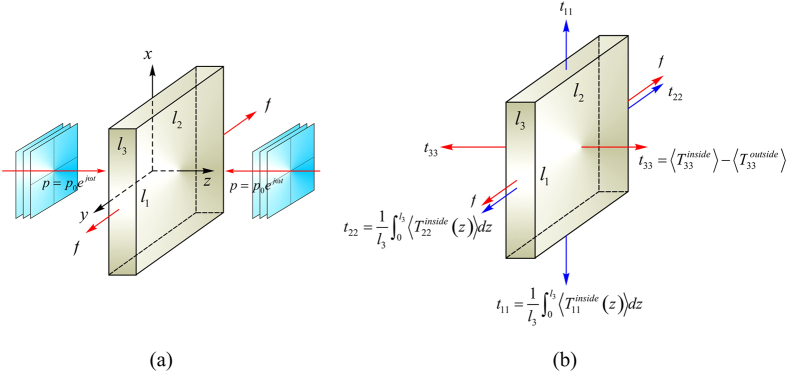
Thin sheet of acoustomechanical soft metamaterial subjected to acoustic wave inputs and mechanical force. (**a**) Two counterpropagating acoustic waves *p* = *p*_0_*e*^*jω*t^ impinge the sheet in the *z*-direction while a mechanical force *f* is exerted on the sheet in the *y*-direction. Under the combined loads of acoustic radiation force and mechanical force, the sheet deforms from initial dimensions (*L*_1_, *L*_2_, *L*_3_) to current dimensions (*l*_1_, *l*_2_, *l*_3_). (**b**) Thin sheet of soft metamaterial subjected to combined equivalent acoustic stresses and mechanical force.

With reference to Fig. 1(a), consider a thin soft material sheet with initial dimensions (*L*_1_, *L*_2_, *L*_3_). The sheet is impinged by two counterpropagating ultrasonic waves *p* = *p*_0_*e*^*jωt*^ in the *z*-direction (thickness direction), *ω* and *p*_0_ being the angular frequency and amplitude of the input sound pressure. Further to acoustic wave inputs, a tensional mechanical force *f* is also exerted on the thin sheet in the *y*-direction. As the two input acoustic fields considered are symmetric with respect to the midplane of the soft material sheet, the system exhibits static deformation because the midplane remains stationary when subjected to two acoustic stresses of equal magnitude but opposing directions. Discontinuity of acoustic impedance at the interface between the soft material and its surrounding medium leads to reflection and refraction of acoustic waves. This process is accompanied by acoustic momentum flux transfer **T** at the interface, thus generating equivalent principal stresses (as shown in Fig. 1(b)) as:


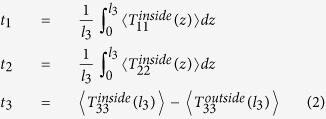


where 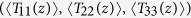 are the acoustic radiation stresses in the principal directions obtained by applying time-averaged manipulation of momentum flux over a cycle, *i.e*., 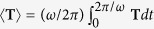, and *l*_3_ is the sheet thickness. The superscripts “inside” and “outside” denote variables related to the inside material and the outside surrounding medium, respectively. Because we assume the acoustic fields only occupy the in-plane area of the soft material sheet, the equivalent stresses *t*_1_ and *t*_2_ only contain the inside material part, while the equivalent stress *t*_3_ contains both the inside material part and the outside surrounding medium part.

To determine the acoustic radiation stresses, one needs first to solve the specific boundary value problem of wave propagation as illustrated in Fig. 1(a). At a prescribed configuration of the soft material sheet, the two counterpropagating ultrasonic waves incident on the material sheet will generate reflected waves and refracted waves (i.e., transmitted waves). Wave propagation both in the material and the surrounding medium is governed by the momentum equation 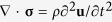 (in Eulerian coordinates). By continuity of acoustic pressure and particle velocity at the interfaces, the acoustic pressure and particle velocity fields inside and outside of the material can be favorably obtained by solving this boundary value problem. Once the acoustic pressure and particle velocity are determined, the acoustic radiation stresses and the equivalent stresses can be calculated by applying Eqs (1) and (2) ; more details are presented in the Supplementary Material.

As above mentioned, acoustic pressure and particle velocity are determined by wave propagation of the two opposing acoustic waves, which is directly dependent upon key parameters like the acoustic impedance of soft material and surrounding medium, acoustic wavelength in soft material, and sheet thickness. Therefore, the equivalent stresses derived on the basis of acoustic pressure and particle velocity rely on these parameters as well.

To facilitate subsequent instability analysis, we constrain the length of the sheet in the *x*-direction to be unchanged and assume the soft material is nearly incompressible, so that its principal stretches (*λ*_1_ = *l*_1_/*L*_1_, *λ*_2_ = *l*_2_/*L*_2_, *λ*_3_ = *l*_3_/*L*_3_) satisfy *λ*_1_ = 1 and 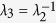. This consideration is enough to capture the fundamental physical nature of tentional acoustomechanical soft metamaterials. We then synthesize the nonlinear elasticity theory of soft materials and the theory of acoustic radiation stresses to establish an acoustomechanical model for such metamaterials. To this end, the Gent model for the Helmholtz free energy function of soft materials is adopted^32^, as:


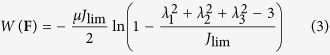


where *μ* and *J*_lim_ are the shear modulus and extension limit of soft material and **F** is the deformation gradient. The Gent model degenerates to the neo-Hookean model when *J*_lim_ approaches infinity. The Cauchy stress is thence given as:


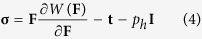


where *p*_*h*_ is a Lagrange multiplier introduced to satisfy the approximation of nearly incompressibility, which is actually the hydrostatic pressure.

Incorporating the mechanical force *f* into the present acoustomechanical problem, we arrive at:


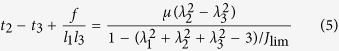


This equation can be rewritten as:


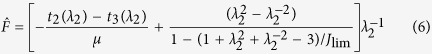


where 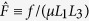 is the normalized mechanical force, which is a function of input acoustic wave amplitude *p*_0_, initial sheet thickness *L*_3_ and stretch *λ*_2_. Equation (6) determines intrinsically the force 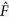 versus stretch *λ*_2_ relation for soft metamaterials. To analyze the acoustomechanical response, we employ the first-order and second-order tangential stiffness of the 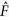 versus *λ*_2_ curve to differentiate the responses in different regimes, as:


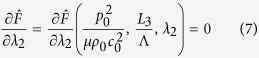



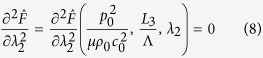


The stability of the acoustomechanical problem considered is determined by Eqs (7) and (8) by ensuring *λ*_2_ has solution within a specified parameter space of 
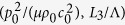
, where 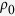 and *c*_0_ are the mass density of and acoustic speed in the soft metamaterial, and 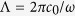 is the acoustic wavelength in the soft metamaterial. The two equations define the phase regions of metamaterial stability: Eq. (7) characterizes the boundary between the s-shaped region and the snapping region while Eq. (8) distinguishes the boundary between the monotonic region and the s-shaped region, as elucidated below.

Adopting the above formulated theoretical model, we calculate the force versus stretch relationship of an acoustomechanical soft metamaterial subjected to the combined external loading of sound pressure and tensile force as shown in Fig. 1. The thin sheet of soft metamaterial is considered to have a thickness comparable to acoustic wavelength Λ and much larger in-plane dimensions. For illustration, because the ultrasonic wavelength in typical hydrogel is approximately 1 mm, the thickness of the sheet can be ~5 mm while its in-plane dimensions and the spot size of acoustic fields can be ~100 mm. Following the custom of nonlinear deformation analysis for soft materials, we consider only homogeneous deformation with principal stretches (*λ*_1_, *λ*_2_, *λ*_3_).

## Results and Discussion

**Figure 2:**
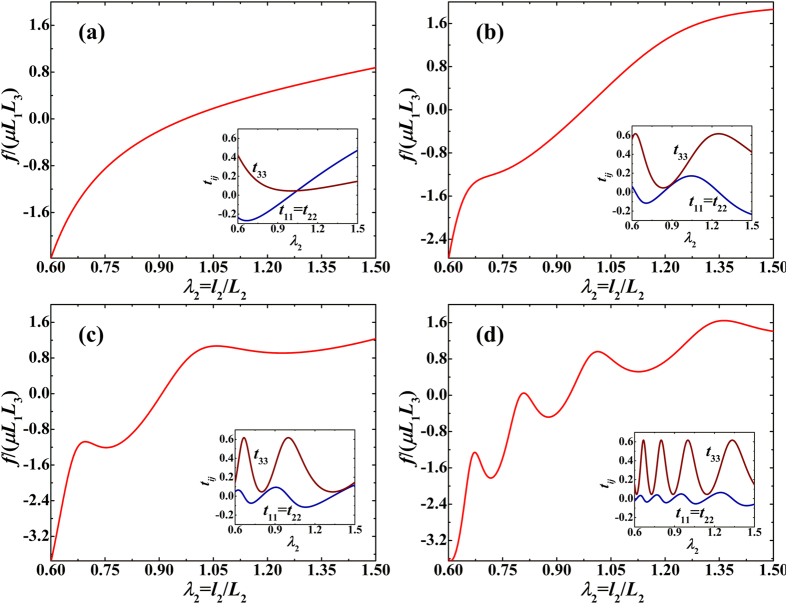
Normalized force versus stretch response of acoustomechanical metamaterial sheet with fixed acoustic input of 
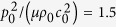
 but different initial sheet thicknesses: (**a**) *L*_3_/Λ = 0.5, (**b**) *L*_3_/Λ = 1.25, (**c**) *L*_3_/Λ = 2.0 and (**d**) *L*_3_/Λ = 4.0. As *L*_3_/Λ is increased, the response sequentially shows monotonic, s-shape, snapping, and multi-snapping phenomena. Inset plots illustrate the variation trends of normalized acoustical radiation stresses with stretch.

Figure 2(a–d) present the normalized force versus stretch curves of the proposed acoustomechanical metamaterial in response to combined acoustical and mechanical loading. Whereas the acoustic wave input is fixed at 
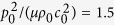
, the normalized initial sheet thickness *L*_3_/Λ is varied as 0.5, 1.25, 2.0 and 4.0. The corresponding normalized equivalent stresses (*i.e*., *t*_11_ = *t*_1_/*μ*, *t*_22_ = *t*_2_/*μ*, *t*_33_ = *t*_3_/*μ*) induced by acoustic wave inputs are also presented as insets. When *L*_3_/Λ is increased, the force versus stretch curves exhibit four different morphological shapes. a) The monotonic shape of Fig. 2(a) where the force monotonically increases as the stretch is increased, with a positive second-order tangential stiffness, and the tendency of the curve is dominated by nonlinear material deformation. b) The s-shape of Fig. 2(b) where the force monotonically increases as the stretch is increased, but with a negative second-order tangential stiffness in the inflexion region. The equivalent stresses play a minor role in creating the inflexion region when *t*_22_–*t*_33_ approaches zero. c) The snapping shape of Fig. 2(c) in which the increase of mechanical force is non-monotonic, accompanied with the appearance of snap-through instability. The first-order tangential stiffness becomes negative when the snap-through instability occurs. In such cases, the equivalent stresses have a larger effect on snapping. d) The multi-snapping shape of Fig. 2(d) where the non-monotonically varying curve exhibits multiple deep snap-through instabilities, caused by the multiple approaching of equivalent stresses *t*_22_ and *t*_33_.

**Figure 3:**
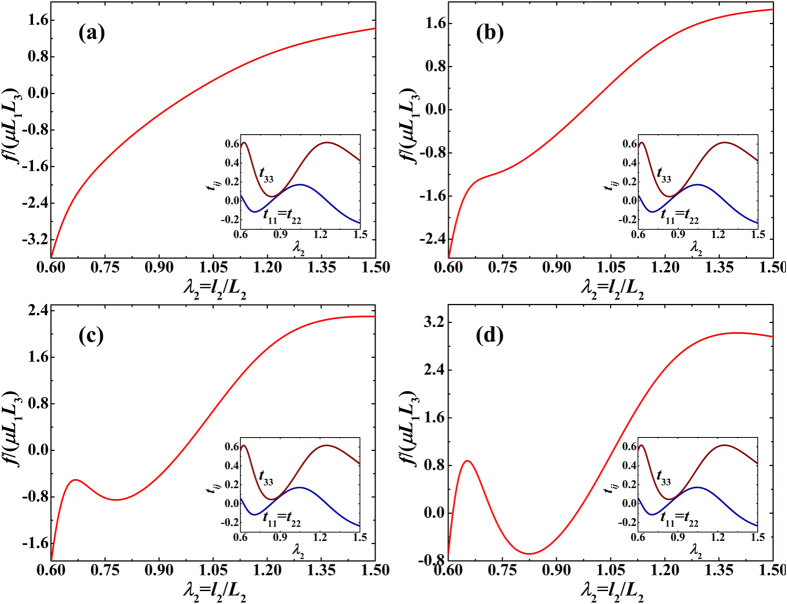
Normalized force versus stretch response of acoustomechanical metamaterial sheet with fixed initial sheet thickness of *L*_3_/Λ = 1.25 but different acoustic inputs: (**a**) 
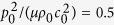
, (**b**) 
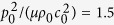
, (**c**) 
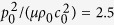
 and (**d**) 
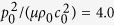
. With increasing acoustic input, the response sequentially displays monotonic, s-shape, snapping, and deep-snapping morphologies. The insert plots illustrate the variation trends of normalized acoustical radiation stresses with stretch.

Next, with the sheet initial thickness fixed at *L*_3_/Λ = 1.25, we vary the acoustic wave input 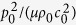 as 0.5, 1.5, 2.5, 4.0 and present in Fig. 3 the resulting force versus stretch curves. Note that the tendency of non-dimensional equivalent stresses relies on *L*_3_/Λ while their magnitudes depend on 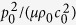. Therefore, the equivalent stresses exhibit the same tendency under different acoustic wave inputs, as shown in the four inserts of Fig. 3. However, increasing the acoustic wave input significantly alters the overall variation trend of the force versus stretch curve, causing monotonic, s-shape, snapping and deep snapping morphologies. In other words, sufficiently large acoustic wave inputs result in snap-through instability and discontinuous deformation of the soft metamaterial. In contrast, under relatively small acoustic wave inputs, the force versus stretch curve exhibits either monotonic tendency or smooth transition. As can be seen from Eq. (6), this arises because large acoustic inputs enhance the effect of 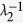 whereas small acoustic inputs weaken the effect of 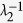.

**Figure 4:**
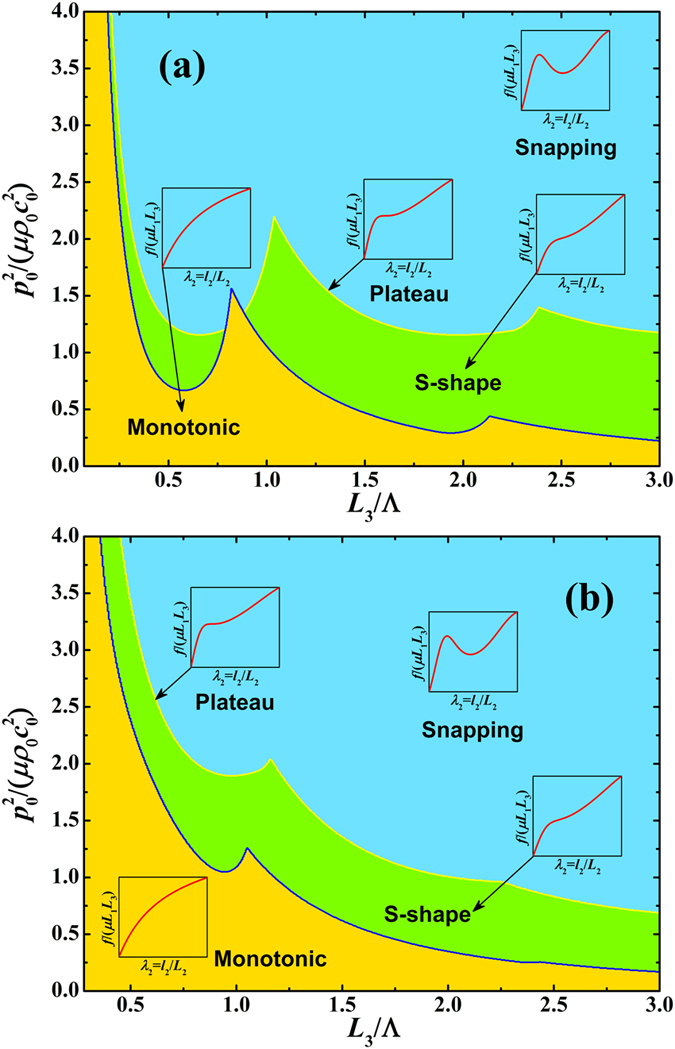
Phase diagrams of acoustomechanical response of thin soft metamaterial sheet under combined mechanical and acoustical loading in the parameter space (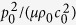, *L*_3_/Λ), showing monotonic, s-shape, plateau, and snapping behaviors for both (**a**) acoustic match (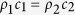) and (**b**) acoustic mismatch (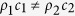) cases.

Equations (7) and (8) demonstrate that the stability of an acoustomechanical soft metamaterial can be divided into three phases, since Eq. (7) divides the positive and negative first-order tangential stiffness while Eq. (8) differentiates the positive and negative second-order tangential stiffness. Upon ensuring the stretch *λ*_2_ has real solution at arbitrarily given values of acoustic input and sheet thickness within the range of (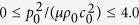, 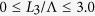), we plot in Fig. 4 the phase diagrams of acoustomechanical responses, for both acoustic match and acoustic mismatch cases. The theoretical force versus stretch curves fall into three main categories: a) monotonic response, b) s-shaped response, and c) non-monotonic snapping or hysteretic response, with representative examples illustrated in the inserts. While a metamaterial sheet with small thickness and low acoustic inputs tends to present monotonic response, that with larger thickness or acoustic inputs tends to exhibit s-shaped and snapping responses (associated with the non-monotonic variation of first-order tangential stiffness). The s-shaped response has positive first-order tangential stiffness throughout the extension history, in contrast to the negative first-order tangential stiffness of snapping response when snap-through instability occurs. The boundary line between the s-shaped and snapping responses is related to the plateau response at which a zero first-order tangential stiffness appears before it becomes again positive. Relative to acoustic mismatch, the acoustic match case exhibits significantly more undulating boundary lines, with the monotonic region even intersecting with the snapping region.

## Conclusions

We have reported a new class of acoustomechanical soft metamaterials with tunable nonlinear mechanical responses by harnessing snap-though instability via programmed acoustic inputs. This new material constructed from homogenous and isotropic soft material acquires unusual properties without resorting to complicated micro/nanoscale architectures required by conventional metamaterials. Moreover, as a fast and non-contact programmable technique, the acoustic inputs can be readily modulated. Therefore, the results of this work enable novel designs of metamaterials with simple architectures and easy programmability via tailored acoustic inputs, promising for a wide variety of potential applications, including tunable tensile devices for wave-guide propagation in optical and acoustic metamaterials, logical gates in microelectromechanical system (MEMS) and microfluidic manipulation devices, etc.

## Additional Information

**How to cite this article**: Xin, F. and Lu, T. Tensional acoustomechanical soft metamaterials. *Sci. Rep*. **6**, 27432; doi: 10.1038/srep27432 (2016).

## Supplementary Material

Supplementary Information
