# Supplementary material for: Tensional acoustomechanical soft metamaterials
Source: Sci Rep. 2016 Jun 6;6:27432. doi: 10.1038/srep27432 (PMC4893735; doi:10.1038/srep27432)
Supplement: Supplementary Information [file srep27432-s1.pdf]

Supplementary information for "Tensional acoustomechanical soft metamaterials"

Fengxian Xin[1,2,*] and Tian Jian Lu[1,2,*]

[1]*State Key Laboratory for Strength and Vibration of Mechanical Structures, Xi'an Jiaotong University, Xi'an 710049, P.R. China*

[2]*MOE Key Laboratory for Multifunctional Materials and Structures, Xi'an Jiaotong University, Xi'an 710049, P.R. China*

Derivation of acoustic fields and equivalent stresses

With reference to Fig. 1 of the main manuscript, consider a thin sheet of soft material immersed in a surrounding medium. A time-harmonic acoustic wave $p(\mathbf{x},t)=p_0e^{-j(\mathbf{k}\cdot\mathbf{x}-\omega t)}$ with wavenumber vector $\mathbf{k}$ is incident on the surface of the sheet from the outside surrounding medium. Due to discontinuity of acoustic impedance at the interfaces, the incident acoustic wave will generate reflection and transmission not only on the inlet surface but also on the outlet surface. In Eulerian coordinates, wave propagation both in the material and the surrounding medium is governed by the momentum equation $\nabla\cdot\boldsymbol{\sigma}=\rho\partial^2\mathbf{u}/\partial t^2$. For ideal fluid-like materials, this equation degrades to the acoustic Helmholtz equation $\rho\partial^2\mathbf{u}/\partial t^2+\nabla p=0$. In such cases, the velocity potentials induced by the incident acoustic wave can be written as:

$$\psi_1(\mathbf{x},t)=\alpha e^{-j\left(\mathbf{k}_1^+\cdot\mathbf{x}-\omega t\right)}+\beta e^{-j\left(\mathbf{k}_1^-\cdot\mathbf{x}-\omega t\right)} \tag{1}$$

$$\psi_2(\mathbf{x},t)=\chi e^{-j\left(\mathbf{k}_2^+\cdot\mathbf{x}-\omega t\right)}+\gamma e^{-j\left(\mathbf{k}_2^-\cdot\mathbf{x}-\omega t\right)} \tag{2}$$

$$\psi_3(\mathbf{x},t)=\vartheta e^{-j\left(\mathbf{k}_3^+\cdot\mathbf{x}-\omega t\right)} \tag{3}$$

where the subscripts 1, 2 and 3 denote the left side medium, the soft material sheet, and the right side medium, while the superscripts + and – are related to positive- and negative-going waves, respectively. The corresponding velocity and pressure fields can be expressed as:

$$\mathbf{u}_i=-\nabla\psi_i,\ p_i=\rho_i\frac{\partial\psi_i}{\partial t}\ (i=1,2,3) \tag{4}$$

---

*fengxian.xin@gmail.com (F.X. Xin); tjlu@mail.xjtu.edu.cn (T.J. Lu)

Continuity of velocity and acoustic pressure at the interfaces dictates:

$$u_{1z} = u_{2z}\big|_{z=0},\ u_{2z} = u_{3z}\big|_{z=l_3} \tag{5}$$

$$p_1 = p_2\big|_{z=0},\ p_2 = p_3\big|_{z=l_3} \tag{6}$$

which can be rewritten in the form:

$$jk_{1z}\alpha e^{-j\left(k_{1x}x+k_{1y}y-\omega t\right)} - jk_{1z}\beta e^{-j\left(k_{1x}x+k_{1y}y-\omega t\right)} = jk_{2z}\chi e^{-j\left(k_{2x}x+k_{2y}y-\omega t\right)} - jk_{2z}\gamma e^{-j\left(k_{2x}x+k_{2y}y-\omega t\right)} \tag{7}$$

$$jk_{2z}\chi e^{-j\left(k_{2x}x+k_{2y}y+k_{2z}l_3-\omega t\right)} - jk_{2z}\gamma e^{-j\left(k_{2x}x+k_{2y}y-k_{2z}l_3-\omega t\right)} = jk_{3z}\vartheta e^{-j\left(k_{3x}x+k_{3y}y+k_{3z}l_3-\omega t\right)} \tag{8}$$

$$j\omega\rho_1\left[\alpha e^{-j\left(k_{1x}x+k_{1y}y-\omega t\right)} + \beta e^{-j\left(k_{1x}x+k_{1y}y-\omega t\right)}\right] = j\omega\rho_2\left[\chi e^{-j\left(k_{2x}x+k_{2y}y-\omega t\right)} + \gamma e^{-j\left(k_{2x}x+k_{2y}y-\omega t\right)}\right] \tag{9}$$

$$j\omega\rho_2\left[\chi e^{-j\left(k_{2x}x+k_{2y}y+k_{2z}l_3-\omega t\right)} + \gamma e^{-j\left(k_{2x}x+k_{2y}y-k_{2z}l_3-\omega t\right)}\right] = j\omega\rho_3\left[\vartheta e^{-j\left(k_{3x}x+k_{3y}y+k_{3z}l_3-\omega t\right)}\right] \tag{10}$$

For simplicity, assuming the same medium occupies the left side and the right side of the sot material sheet, *i.e.*, $\rho_1 = \rho_3$ and $k_1 = k_3$, one has:

$$\beta = \frac{\alpha e^{jk_{2z}l_3}\left(\rho_2^2k_{1z}^2 - \rho_1^2k_{2z}^2\right) + \alpha e^{-jk_{2z}l_3}\left(\rho_1^2k_{2z}^2 - \rho_2^2k_{1z}^2\right)}{e^{jk_{2z}l_3}\left(\rho_1k_{2z} + \rho_2k_{1z}\right)^2 - e^{-jk_{2z}l_3}\left(\rho_1k_{2z} - \rho_2k_{1z}\right)^2} \tag{11}$$

$$\chi = \frac{2\alpha\rho_1k_{1z}e^{jk_{2z}l_3}\left(\rho_2k_{1z} + \rho_1k_{2z}\right)}{e^{jk_{2z}l_3}\left(\rho_1k_{2z} + \rho_2k_{1z}\right)^2 - e^{-jk_{2z}l_3}\left(\rho_1k_{2z} - \rho_2k_{1z}\right)^2} \tag{12}$$

$$\gamma = \frac{2\alpha\rho_1k_{1z}e^{-jk_{2z}l_3}\left(\rho_1k_{2z} - \rho_2k_{1z}\right)}{e^{jk_{2z}l_3}\left(\rho_1k_{2z} + \rho_2k_{1z}\right)^2 - e^{-jk_{2z}l_3}\left(\rho_1k_{2z} - \rho_2k_{1z}\right)^2} \tag{13}$$

$$\vartheta = \frac{4\alpha\rho_1\rho_2k_{1z}k_{2z}e^{jk_{1z}l_3}}{e^{jk_{2z}l_3}\left(\rho_1k_{2z} + \rho_2k_{1z}\right)^2 - e^{-jk_{2z}l_3}\left(\rho_1k_{2z} - \rho_2k_{1z}\right)^2} \tag{14}$$

Next, assume two counter-propagating acoustic waves $p_L\left(z,t\right) = p_0e^{-j\left(k_{1z}z-\omega t\right)}$ and $p_R\left(z,t\right) = p_0e^{j\left(k_{1z}\left(z-l_3\right)+\omega t\right)}$ are normally incident on the soft material sheet (Fig. 1) so that the two input acoustic fields are symmetric with respect to its midplane. As a result, the system exhibits static deformation because the midplane remains stationary when subjected to two acoustic stresses of equal magnitude but opposing directions. The acoustic pressure and velocity can thus be expressed as:

$$p_1 = j\omega\rho_1\alpha e^{j\omega t}\left[e^{-jk_{1z}z} + e^{jk_{1z}z}\frac{j\sin\left(k_{2z}l_3\right)\left(\rho_2^2k_{1z}^2 - \rho_1^2k_{2z}^2\right) + 2\rho_1\rho_2k_{1z}k_{2z}}{2\rho_1\rho_2k_{1z}k_{2z}\cos\left(k_{2z}l_3\right) + j\left(\rho_1^2k_{2z}^2 + \rho_2^2k_{1z}^2\right)\sin\left(k_{2z}l_3\right)}\right] \quad (15)$$

$$u_{1z} = jk_{1z}\alpha e^{j\omega t}\left[e^{-jk_{1z}z} - e^{jk_{1z}z}\frac{j\sin\left(k_{2z}l_3\right)\left(\rho_2^2k_{1z}^2 - \rho_1^2k_{2z}^2\right) + 2\rho_1\rho_2k_{1z}k_{2z}}{2\rho_1\rho_2k_{1z}k_{2z}\cos\left(k_{2z}l_3\right) + j\left(\rho_1^2k_{2z}^2 + \rho_2^2k_{1z}^2\right)\sin\left(k_{2z}l_3\right)}\right] \quad (16)$$

$$p_2 = \frac{2\omega\alpha\rho_1\rho_2k_{1z}e^{j\omega t}\left[\rho_2k_{1z}\sin\left(k_{2z}\left(z-l_3\right)\right) - \rho_2k_{1z}\sin\left(k_{2z}z\right) + j\rho_1k_{2z}\cos\left(k_{2z}\left(z-l_3\right)\right) + j\rho_1k_{2z}\cos\left(k_{2z}z\right)\right]}{2\rho_1\rho_2k_{1z}k_{2z}\cos\left(k_{2z}l_3\right) + j\left(\rho_1^2k_{2z}^2 + \rho_2^2k_{1z}^2\right)\sin\left(k_{2z}l_3\right)} \quad (17)$$

$$u_{2z} = \frac{2\alpha\rho_1k_{1z}k_{2z}e^{j\omega t}\left[\rho_1k_{2z}\sin\left(k_{2z}\left(z-l_3\right)\right) + \rho_1k_{2z}\sin\left(k_{2z}z\right) + j\rho_2k_{1z}\cos\left(k_{2z}\left(z-l_3\right)\right) - j\rho_2k_{1z}\cos\left(k_{2z}z\right)\right]}{2\rho_1\rho_2k_{1z}k_{2z}\cos\left(k_{2z}l_3\right) + j\left(\rho_1^2k_{2z}^2 + \rho_2^2k_{1z}^2\right)\sin\left(k_{2z}l_3\right)} \quad (18)$$

$$p_3 = j\omega\rho_1\alpha e^{j\omega t}\left[e^{jk_{1z}(z-l_3)} + e^{-jk_{1z}(z-l_3)}\frac{j\sin\left(k_{2z}l_3\right)\left(\rho_2^2k_{1z}^2 - \rho_1^2k_{2z}^2\right) + 2\rho_1\rho_2k_{1z}k_{2z}}{2\rho_1\rho_2k_{1z}k_{2z}\cos\left(k_{2z}l_3\right) + j\left(\rho_1^2k_{2z}^2 + \rho_2^2k_{1z}^2\right)\sin\left(k_{2z}l_3\right)}\right] \quad (19)$$

$$u_{3z} = jk_{1z}\alpha e^{j\omega t}\left(-e^{jk_{1z}(z-l_3)} + e^{-jk_{1z}(z-l_3)}\frac{j\sin\left(k_{2z}l_3\right)\left(\rho_2^2k_{1z}^2 - \rho_1^2k_{2z}^2\right) + 2I\rho_1\rho_2k_{1z}k_{2z}}{2\rho_1\rho_2k_{1z}k_{2z}\cos\left(k_{2z}l_3\right) + j\left(\rho_1^2k_{2z}^2 + \rho_2^2k_{1z}^2\right)\sin\left(k_{2z}l_3\right)}\right) \quad (20)$$

Once the pressure and velocity are known, the acoustic radiation stress tensors both inside and outside the material can be calculated by time-averaging the corresponding variables, as:

$$\left\langle \mathbf{T}^{outside}\right\rangle \equiv \left[\frac{\left\langle p_1^2\right\rangle}{2\rho_1c_1^2} - \frac{\rho_1\left\langle \mathbf{u}_1\cdot\mathbf{u}_1\right\rangle}{2}\right]\mathbf{I} + \rho_1\left\langle \mathbf{u}_1\otimes\mathbf{u}_1\right\rangle \quad (21)$$

$$\left\langle \mathbf{T}^{inside}\right\rangle \equiv \left[\frac{\left\langle p_2^2\right\rangle}{2\rho_2c_2^2} - \frac{\rho_2\left\langle \mathbf{u}_2\cdot\mathbf{u}_2\right\rangle}{2}\right]\mathbf{I} + \rho_2\left\langle \mathbf{u}_2\otimes\mathbf{u}_2\right\rangle \quad (22)$$

where the subscripts "1, 2" denote the outside medium and the inside medium, respectively. As shown in Fig. 2(b), the equivalent stresses can be obtained by homogenizing the acoustic radiation stresses, as:

$$t_1 = \frac{1}{l_3}\int_0^{l_3}\left\langle T_{11}^{inside}\left(z\right)\right\rangle dz,\ t_2 = \frac{1}{l_3}\int_0^{l_3}\left\langle T_2^{inside}\left(z\right)\right\rangle dz,\ t_3 = \left\langle T_{33}^{inside}\left(l_3\right)\right\rangle - \left\langle T_{33}^{outside}\left(l_3\right)\right\rangle \quad (23)$$

where l_3 is the thickness of the sheet, ($\left\langle T_{11}\left(z\right)\right\rangle$, $\left\langle T_{22}\left(z\right)\right\rangle$, $\left\langle T_{33}\left(z\right)\right\rangle$) are the acoustic radiation stresses in the principal directions obtained by applying time-averaged manipulation of momentum flux over a cycle, *i.e.*, $\left\langle \mathbf{T}\right\rangle = \left(\omega/2\pi\right)\int_0^{2\pi/\omega}\mathbf{T}dt$, and ($t_1$, t_2, t_3) are the corresponding equivalent stresses, as shown in Fig. 1(b). The superscripts "inside" and "outside" denotes

variables related to the inside material and the outside surrounding medium, respectively. If the acoustic fields are assumed to only occupy the in-plane region of the soft material sheet, the equivalent stresses t_1 and t_2 only contain the inside material part, while the equivalent stress t_3 contains both the inside material part and the outside surrounding medium part. These equivalent stresses are the resultant stresses that cause large deformation in the soft material.
